# Genome sequence of a polerovirus isolated from wild oat in Australia

**DOI:** 10.1128/mra.00825-23

**Published:** 2023-12-22

**Authors:** N. Nancarrow, W. M. Kinoti, S. K. Lam, B. Rodoni, P. Trębicki

**Affiliations:** 1 School of Agriculture, Food and Ecosystem Sciences, The University of Melbourne, Parkville, Victoria, Australia; 2 Agriculture Victoria, Grains Innovation Park, Horsham, Victoria, Australia; 3 Agriculture Victoria, AgriBio Centre, Bundoora, Victoria, Australia; 4 School of Applied Systems Biology, La Trobe University, Bundoora, Victoria, Australia; 5 Applied BioSciences, Macquarie University, Sydney, New South Wales, Australia; Katholieke Universiteit Leuven, Leuven, Belgium

**Keywords:** cereal, cereal yellow dwarf virus, polerovirus, wild oat (*Avena fatua*)

## Abstract

We present the genome sequence of a polerovirus (family Solemoviridae) isolated from wild oat (*Avena fatua*) in Australia. The genome sequence consists of 5,631 nucleotides and shares 87% nucleotide identity with its closest relative, cereal yellow dwarf virus RPV isolate 010 (GenBank accession number EF521830).

## ANNOUNCEMENT

Poleroviruses, such as cereal yellow dwarf virus RPV (CYDV RPV), maize yellow dwarf virus RMV (MYDV RMV), and barley virus G (BVG), belong to the family Solemoviridae. Along with the luteoviruses barley yellow dwarf virus PAV (BYDV PAV) and barley yellow dwarf virus MAV (BYDV MAV) (family Tombusviridae), these poleroviruses infect a wide range of cereal and grass hosts in Australia and worldwide. In October 2020, a tiller was collected from a wild oat (*Avena fatua*) plant (33WO) from alongside a wheat field located near Kewell, Victoria, Australia. The plant displayed red-yellow leaf symptoms. When tested by tissue blot immunoassay, the tiller tested negative for BYDV PAV, BYDV MAV and CYDV RPV but positive using an antibody (DSMZ AS-0227/1) that detects a range of luteovirus and/or polerovirus species (1). Total RNA extracted from the tiller using the RNeasy Plant Mini Kit (Qiagen) with modified lysis buffer (2, 3) was positive by RT-PCR for CYDV RPV and negative for BYDV PAV, MYDV RMV (4), and BVG (5). A high-throughput sequencing (HTS) library was prepared using an NEBNext Ultra II RNA Library Prep Kit for Illumina (NEB) and was sequenced on a NovaSeq 6000 (Illumina) platform. Raw paired-end HTS reads (average length 152 bp) were trimmed using fastp v0.20.0 (6). *De novo* assembly of the resulting 24,230,232 trimmed reads with the SPAdes genome assembler v3.15.3 (7) generated 29,329 contigs of between 128 and 21,989 nucleotides (nt) in length. BLASTn analysis of the resulting contigs revealed only one large virus-like contig (5,631 nt), which was most similar to CYDV RPV, sharing 86%–87% nt identity with the CYDV RPV isolate 010 (EF521830) and the CYDV RPV reference sequence (NC_004751). The genome sequence was confirmed by mapping 45,540 trimmed HTS reads to it with Bowtie2 v2.4.5 (8), with 100% coverage and an average coverage depth of 1,151×. The genome has a structure typical of poleroviruses (9) and a GC content of 50%. BLASTx analysis of the seven individual major open reading frame (ORF) sequences showed that all had the highest similarity to CYDV RPV, with ORFs 3a, 3, 4, and 3–5 sharing 91%, 97%, 92%, and 90% amino acid (aa) similarity, respectively, with the CYDV RPV reference sequence (NC_004751) (Table 1). However, the highest similarity that ORFs 0, 1, and 1–2 shared with CYDV RPV aa sequences on NCBI GenBank was 66% (NP_840020), 75% (ABP68673), and 84% (ABP68672), respectively. The genome sequence was further confirmed using primers that amplified overlapping PCR amplicons across the genome, which were then directly Sanger sequenced. The sequence (33WO) was deposited in GenBank (LC765997). Additionally, one wild oat and three wheat (*Triticum aestivum*) plants collected from two other fields in the same week as sample 33WO also tested positive for this isolate by RT-PCR using the primers 5′-CCAGCCTTCTTTAAGCGCTCTT-3′ and 5′-GCTTTCCACAGCCAACTCGTTA-3′, which targeted the P0 gene, and were confirmed by Sanger sequencing. Further analysis is required to clarify the prevalence of this polerovirus isolate and its relationship with CYDV RPV.

**TABLE 1:** Genome nucleotide identity and amino acid similarities (%) between the polerovirus isolated from wild oat in Australia and the virus species most closely related to it

Polerovirus species	Accession number	Genome (nt)	ORF0 (aa)	ORF1 (aa)	ORF1–2 (aa)	ORF3a (aa)	ORF3 (aa)	ORF4 (aa)	ORF3–5 (aa)
Cereal yellow dwarf virus RPV	NC_004751	86.2	66.0	73.8	83.6	91.1	97.1	92.2	90.0
Cereal yellow dwarf virus RPS	NC_002198	84.3	38.7	53.3	68.3	75.6	90.7	88.2	82.7
Wheat yellow dwarf virus GPV	NC_012931	80.4	39.8	55.0	69.4	86.7	78.4	81.0	74.0

## Data Availability

The genome sequence of the isolate described here was deposited in DDBJ/EMBL/GenBank under accession number LC765997. Raw sequence data were deposited in the SRA under BioProject number PRJDB16503 and under BioSample number SAMD00639105.
